# Data reduction for serial crystallography using a robust peak finder

**DOI:** 10.1107/S1600576721007317

**Published:** 2021-09-13

**Authors:** Marjan Hadian-Jazi, Alireza Sadri, Anton Barty, Oleksandr Yefanov, Marina Galchenkova, Dominik Oberthuer, Dana Komadina, Wolfgang Brehm, Henry Kirkwood, Grant Mills, Raphael de Wijn, Romain Letrun, Marco Kloos, Mohammad Vakili, Luca Gelisio, Connie Darmanin, Adrian P. Mancuso, Henry N. Chapman, Brian Abbey

**Affiliations:** a ARC Centre of Excellence in Advanced Molecular Imaging, La Trobe Institute for Molecular Sciences, La Trobe University, Melbourne, Australia; b Australian Nuclear Science and Technology Organisation (ANSTO), Australia; c European XFEL, Holzkoppel 4, 22869 Schenefeld, Germany; d Center for Free-Electron Laser Science, Deutsches Elektronen-Synchrotron (DESY), Notkestrasse 85, 22607 Hamburg, Germany; e Department of Chemistry and Physics, La Trobe Institute for Molecular Science, La Trobe University, Melbourne, Victoria, Australia; f Department of Physics, Universität Hamburg, Luruper Chaussee 149, 22761 Hamburg, Germany; g The Hamburg Centre for Ultrafast Imaging, Luruper Chaussee 149, 22761 Hamburg, Germany

**Keywords:** data reduction, serial crystallography, robust statistics, Bragg peak finding

## Abstract

A peak-finding algorithm for serial crystallography (SX) data analysis based on the principle of ‘robust statistics’ has been developed. Methods which are statistically robust are generally more insensitive to any departures from model assumptions and are particularly effective when analysing mixtures of probability distributions. For example, these methods enable the discretization of data into a group comprising inliers (*i.e.* the background noise) and another group comprising outliers (*i.e.* Bragg peaks). Our robust statistics algorithm has two key advantages, which are demonstrated through testing using multiple SX data sets. First, it is relatively insensitive to the exact value of the input parameters and hence requires minimal optimization. This is critical for the algorithm to be able to run unsupervised, allowing for automated selection or ‘vetoing’ of SX diffraction data. Secondly, the processing of individual diffraction patterns can be easily parallelized. This means that it can analyse data from multiple detector modules simultaneously, making it ideally suited to real-time data processing. These characteristics mean that the robust peak finder (RPF) algorithm will be particularly beneficial for the new class of MHz X-ray free-electron laser sources, which generate large amounts of data in a short period of time.

## Abstract 2

This article focuses on the challenges of hit finding and data reduction in serial crystallography (SX). An effective and reliable Bragg-peak-finding method, called robust peak finder (RPF), has been developed. RPF is based on the principle of robust statistics and can be used for SX data analysis.

## 1. Introduction   

X-ray crystallography is one of the most important tools in structural biology, responsible for over 80% of the biomol­ecular structures solved today and deposited in the Protein Data Bank (Berman *et al.*, 2003 ▸). The first hard X-ray free-electron lasers (XFELs) capable of high-resolution serial femtosecond crystallography (SFX) measurements only came online in 2009 (Chapman *et al.*, 2011 ▸). The recent new methodological development of serial crystallography (SX) has brought new capabilities for obtaining time-resolved and static structures of macromolecules, potentially outrunning radiation damage and without the need for cryogenic cooling. First demonstrated at XFEL facilities, serial crystallography involves the collection of single-snapshot diffraction patterns from individual crystals, at rates that are only limited by the frequency of the X-ray pulses or the frame rate of detectors. Many of the new XFEL facilities which began operation within the past few years have data acquisition rates far higher than those achieved with the first generation of XFELs. We are now in an era of ultra-high-throughput experiments that can track the evolution of macromolecular systems as they undergo reactions or responses to various perturbations (Mills *et al.*, 2020 ▸). Serial crystallography experiments performed at facilities such as the European XFEL (EuXFEL) generate massive data sets that can be as large as 1 petabyte (10^15^ bytes) per experiment (Wiedorn *et al.*, 2018 ▸). The rapid generation of this amount of data necessitates the development of suitable facilities to be able to manage it. This includes appropriate data storage, networking and data analysis platforms. In order to address these issues there is an urgent need to develop efficient and robust solutions for processing and analysing data. The goal is to be able to filter data sets, by rejecting data that are unusable or do not contain any useful information, whilst preserving all images which contain any signal produced by interaction of the beam with the sample. This need has motivated the current effort to develop a robust and efficient method for detecting Bragg peaks which can then be deployed to reduce the size of the data set obtained during SX experiments.

The serial crystallography method comprises many steps (Darmanin *et al.*, 2016 ▸). The sample is delivered via fixed target holders or as a continuous stream of liquid to the region of interaction with the X-ray beam (Schlichting, 2015 ▸; Berntsen *et al.*, 2019 ▸). Diffraction frames are recorded for every individual X-ray pulse or repetition cycle of the detector, regardless of whether a crystal is actually within the X-ray beam or not. If the X-ray beam interacts with a crystal, the recorded diffraction pattern may contain discrete Bragg peaks formed via the crystal. Otherwise, the Bragg peaks are absent and only the diffuse signal produced via interaction with the jet stream is detected. This scattering usually gives rise to a diffuse pattern that is often treated as a background that is independent of the crystal diffraction (Chapman *et al.*, 2017 ▸; Hajdu, 2017 ▸). By identifying and discriminating those detector frames that contain Bragg peaks (known as ‘hits’), and removing any frames which only contain background scatter, the volume of data can usually be significantly reduced; this process is known as ‘hit finding’. This task of identifying hits is accomplished by programs that determine the presence and locations of individual Bragg peaks, collectively referred to as ‘peak finders’. The identification of individual Bragg peaks from 2D diffraction data is referred to as ‘peak finding’ (Schlichting, 2015 ▸).

One approach to reducing the size of the data set is to avoid storing any data frames that are not hits. This involves real-time monitoring of the experiment in order to determine when a particular frame of data should be read from the detector and moved to more permanent storage. Successful peak finders for online monitoring systems, such as* OnDA * (Mariani *et al.*, 2016 ▸), *CASS* (Foucar *et al.*, 2012 ▸), *CCTBX* (Grosse-Kunstleve *et al.*, 2002 ▸), *Hummingbird* (Daurer *et al.*, 2016 ▸) (for single-particle imaging), and Linac Coherent Light Source (LCLS) *AMI* and *Psocake* (Thayer *et al.*, 2017 ▸) (a graphical user interface for finding Bragg peaks), use peak-finding algorithms to provide live feedback about the data quality and number of hits to the experimental team. This information is then used to optimize the measurements and determine the viability of the sample. Even though peak-finding methods have been used successfully previously, parameters often need to be optimized during the experiment before they can work effectively. This limits their reliability and effectiveness in the context of online data processing, and has motivated the development of a more robust approach which is the subject of this paper. Parameter optimization of peak-finding algorithms frequently involves multiple attempts leading to duplicate sets of analysed data which require even larger data storage. This also leads to a significant waste of experimental beam time and hence negatively impacts the costs of running the facility. Parameter optimization is also time consuming, which then limits the ability of these algorithms to provide real-time feedback and often leads to uncertainty over whether the best parameters have actually been selected. The development of a more robust approach to peak finding would allow for a common set of parameters to be employed throughout the experiment and between different samples. It would lower the barrier to entry for non-expert users of SFX and allow the beamline to enact a ‘veto’ system to dramatically reduce the final data volume. In this paper, we report on our recent development of a new approach to robust hit finding and evaluate its performance using an explicit mathematical foundation for peak selection. Our algorithm employs a robust statistical framework, so we refer to it henceforth as the robust peaking finder (RPF) algorithm.

The structure of the paper is as follows. In Sections 2 and 3, we review current peak-finder methods to provide a context for the present work. In Section 4, the methodology of the proposed robust peak finder is introduced. The algorithm is applied to a number of different data sets collected under different experimental conditions to check its performance; the results are reported in Section 5. The reliability and accuracy of the robust peak-finding algorithm is then assessed with respect to data reduction and compared with the current state of the art. We conclude with a discussion of the benefits of using the algorithm in terms of online SX data monitoring.

## 2. Background   

Most of the current methods used to perform peak finding and data reduction in serial crystallography are heuristic methods. An example is reported by Li & Zatsepin (2018 ▸), who uses a simple global threshold to separate the background signal from the Bragg peaks. This approach is straightforward but its effectiveness is often highly dependent on the choice of input parameters. In this paper we propose a peak-finding method that does not depend on the global threshold in order to differentiate the Bragg peak intensities.

Among the current suite of hit-finding algorithms there are those that use statistical methods to find a threshold that separates Bragg peaks from the background (Barty *et al.*, 2014 ▸; Parkhurst *et al.*, 2016 ▸; Hadian-Jazi *et al.*, 2017 ▸). With these algorithms it is typically assumed that a geometric model (*e.g.* a single scalar value or a four-parameter model plane in three dimensions, normally a linear ramp of intensity values fitted to a 2D array) can be used to represent the intensity values of pixels belonging to the background (referred to here as inliers) in the immediate vicinity of a peak. This model is then used to separate the background from Bragg peaks (which we term ‘outliers’ since they are excluded from the model of the background). The accurate estimation of model parameters is key to the success of these methods as any error in the assumed model for the background prevents the successful separation of inliers and outliers. As such, the model parameters should be calculated on the basis of a characterization of the inliers, avoiding any dependence on the distribution of outliers. However, the challenge is that the inliers are initially unknown. For example, in a given diffraction pattern, it is not known prior to analysis where the Bragg peaks will be located as this is highly dependent on the crystal packing and the crystal orientation relative to the X-ray beam. An essential requirement is thus that the statistics used to define the model need to be robust with respect to the presence of outliers (Bragg peaks).

Different statistical approaches have varying degrees of robustness with respect to outliers depending on their associated probability distribution (Huber, 2009 ▸). For example, some statistical measures may depend on the number of Bragg peaks or their intensities. Consider a set of intensity values *X* = {*x*_*i*_} of *N* pixels of a diffraction pattern distributed according to a Poisson probability density function. There are three common statistics models for the background: the sample mean, 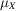 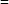 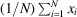; the sample variance, 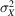 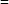 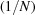 × 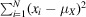; and the median, 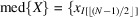 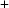 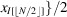 (where *I* is the set of indices that sorts *X* and 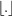 is the floor). Upon manually increasing one of the values in *X* towards infinity (to make it an outlier mimicking a Bragg peak), the variance increases the most. The average increases as well but not as rapidly, whilst the median does not change. The median is referred to as a robust statistic since it disregards the one outlier, whilst the average or variance are conventionally called non-robust statistics (Huber, 2009 ▸). Therefore the benefit of using a robust statistical method is that it results in a model that fits the background irrespective of the number and intensities of Bragg peaks.

There are a number of different software packages available for SX data analysis. One of the most commonly used is called *Cheetah* (Barty *et al.*, 2014 ▸). The hit-finding algorithm peakfinder8 (PF8) from *Cheetah* is frequently employed in other hit-finding software such as *OnDA* (Mariani *et al.*, 2016 ▸) and *CrystFEL* (White *et al.*, 2012 ▸, 2016 ▸). PF8 uses non-robust statistics along with careful algorithmic outlier removal to detect the location of Bragg peaks. PF8 is similar to adaptive MeanShift (Comaniciu & Meer, 2002 ▸; Comaniciu *et al.*, 2001 ▸), which is an expectation-maximization algorithm that iteratively updates the model prior to the detection of outliers.

PF8 starts by modelling the pixel intensities on one resolution ring around the centre of the diffraction pattern (with the set of intensities of all pixels denoted by *X*), using a single scalar value which is the average, μ_*X*_, and a scale which defines the Gaussian noise (σ_*X*_). The method used to analyse this data subset is a fit-and-remove outlier deletion algorithm that segments outliers from inliers. We define a signal-to-noise ratio (SNR) to quantify the quality of segmentation of outliers and inliers. There are many possible definitions for the SNR; here we propose to use the statistical separability (Wilkinson *et al.*, 1988 ▸; Hadian-Jazi *et al.*, 2015 ▸). We define the SNR of the segmentation of the distributions of inliers and outliers in terms of a common measure of the statistical separability of two distributions, given by 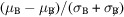, where inliers are denoted by set B and outliers by set 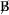 and 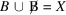. Here μ_B_ and 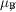 are the sample means of the distributions of the pixels of inliers and outliers, respectively, and σ_B_ and 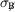 the corresponding standard deviations of those distributions. The SNR is used to measure the quality of every peak. PF8 also uses this definition for SNR, which requires the correct estimation of μ_B_ and σ_B_ – a crucial task in order to ensure its successful implementation.

Given a minimum acceptable SNR τ, the fit-and-remove algorithm in PF8 works as follows: A threshold is defined as *T* = μ_*X*_ + τσ_*X*_. Those pixels above the threshold are removed from *X*, μ_*X*_ and σ_*X*_ are recalculated, and the threshold is updated accordingly. The algorithm repeats this process five times. This process produces a threshold for each resolution shell. This approach assumes that the background has no azimuthal dependence, *e.g.* has been corrected for polarization. The calculation of the threshold uses intensities of all of the pixels (*X*) which includes both inliers and outliers. Afterwards, the algorithm estimates the average and the standard deviation of local background pixels. The SNR is also calculated in the presence of outliers which were not identified earlier and hence the process is not robust. This means that the final SNR is compared with the initial SNR before removing outlier pixels from *X*. PF8 calculates an SNR for each Bragg peak and reports the location and intensity of those above the set SNR threshold, τ.

Another robust background modelling method was included as part of the *DIALS* analysis software package (Parkhurst *et al.*, 2016 ▸). The mathematical approach used to develop that method is similar to PF8 in terms of model fitting. However, it uses a Huber estimator (Huber, 2009 ▸) to analyse fitting errors normalized by the standard deviation of the data tuned to capture 95% of inliers. This is in contrast to PF8 which simply removes those points that have values more than a few standard deviations of the mean of the distribution. We argue that the result of fitting without excluding outliers in estimating the background model parameters depends on the number of outliers and how they are spread above the minimum acceptable threshold. By contrast, the RPF algorithm, discussed in Section 4, uses an optimization technique that models the density of inliers and uses it to define the minimum acceptable threshold independent of the density of outliers, making it a robust model.

## 3. Peak finding   

Some conditions in serial crystallography can make modelling with non-robust methods particularly challenging. One is when a diffraction pattern includes a large number of Bragg peaks, which means that the non-robust statistics approaches (such as the fit-and-remove method described above) have to deal with many outliers. This problem becomes more apparent in dealing with detectors with an increasingly high pixel density, which requires the method to re-bin the data and/or analyse the background within small regions of the image. For example, when two Bragg peaks, each covering six pixels, are located within a window of size 16 × 16 pixels and their centres are just eight pixels apart diagonally, the model fitting method needs to be able to cope with 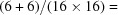 4.7% outliers within that window. One solution would be to simply increase the size of the window. However, this reduces the speed of the algorithm and fails when dealing with large numbers of Bragg peaks. In Fig. 2 below, we show that the probability of correctly detecting a Bragg peak with a non-robust method such as PF8 dramatically decreases when the percentage of outliers is around 5%, in contrast to our RPF model which stays consistent without changing any parameters.

Non-robust approaches to peak finding can fail when the intensities of Bragg peaks are very low, close to the level of the background. This situation is particularly common at higher resolutions where Bragg peaks are not easily distinguishable from the shot noise. Nonlinear noise or unusual detector response characteristics can also reduce the SNR of Bragg peaks calculated using non-robust methods and can artificially raise the threshold for the background, causing weak peaks to be ignored.

The peak finder presented in this paper, based on the principles of robust statistics, disregards the density of outliers when constructing a model for the inliers. In Section 5, we show that the proposed peak finder is able to reliably detect a larger proportion of weaker peaks, leading to more accurate indexing at higher resolution. The details of the proposed method are described in Section 4. However, in order to highlight the drawbacks of non-robust methods and show how robust methods can improve the performance of peak finding in the above situations, we present some examples of analysing simulated data sets. These data sets consisted of an experimental background but simulated Bragg reflections whereby we could arbitrarily set the SNR.

In the first example, we used images obtained from an SX experiment data set performed using the SPB/SFX instrument (Mancuso *et al.*, 2019 ▸). The liquid sample (lysozyme crystals suspended in a buffer) was delivered using a 3D-printed gas dynamic virtual nozzle (GDVN) (Knoška *et al.*, 2020 ▸). More details about the experiment and the data set are given in Section 5.2. We manually chose images that did not include any Bragg peaks and then added a number of simulated peaks to them. The intensity of these peaks was chosen to be close to the threshold set by the minimum acceptable SNR of τ = 6. This threshold value was chosen to match the typical width of Bragg peaks that are measured during SFX experiments using the AGIPD detector at the EuXFEL, which normally vary between 1 to 6 pixels in width. Since initially there are no Bragg peaks in these patterns, we used the mean of the data as the background model value and the standard deviation as the noise scale, this determines the threshold used for the simulation.

**Figure 1:**
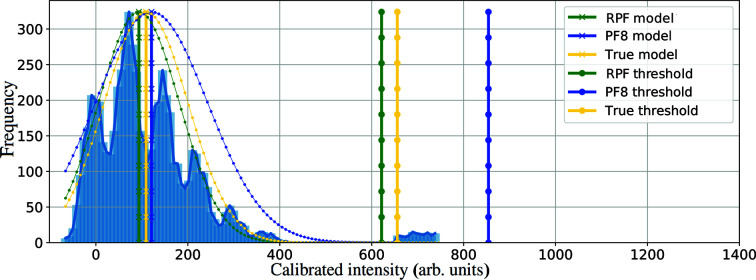
The outliers are included in the histogram (frequency versus calibrated intensity) to show the cut-off threshold of the robust and non-robust algorithms and contrast how each approach models the background. In this example, to compare robust and non-robust methods (see text), PF8 misses some of the weaker Bragg peaks because of its sensitivity to the presence of outliers in estimating the background model.

The number of outliers (peaks) and their values were the input for the simulation. The inlier cut-off thresholds calculated with robust and non-robust statistics approaches are reported and shown in Fig. 1 ▸. The histogram in Fig. 1 ▸ has two distinct distributions: to the left is the intensity of background pixels and to the right is a uniformly distributed set of synthetic Bragg peak pixel intensities. The SNR of these peaks was set to be between 6 and 6 + *w*. In Fig. 1 ▸, *w* = 2 and 2% of data are outliers. Because the non-robust statistics approach (PF8) includes outliers when calculating the noise scale for the background intensities, its performance is impacted when detecting weak reflections. Consequently PF8 could not calculate the true mean, artificially raising the cut-off threshold and causing the algorithm to miss some of the weaker peaks.

**Figure 2:**
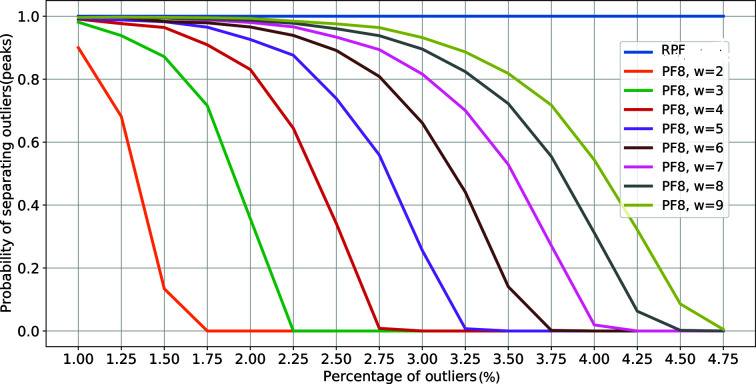
Probability of correctly identifying Bragg peaks as a function of the density of Bragg peaks in the diffraction pattern (‘percentage of outliers’). As the number of Bragg peaks increases the performance of the PF8 algorithm decreases. This is compensated for as the spread in Bragg peak intensities (higher *w*) increases, *i.e.* for the same percentage of outliers, a higher *w* leads to an improvement in the probability of correctly identifying Bragg peaks. The RPF model (blue line) is consistent in its detection irrespective of outlier positioning.

The success of PF8 is dependent on the density of outliers. We evaluated this by varying the percentage of pixels belonging to Bragg peaks and by changing the value of *w*. We repeated these tests 10 000 times; the average of the percentage of correctly labelled outliers is shown in Fig. 2 ▸. In this figure, the predicted spot positions identified using RPF and PF8 are cross-checked using the known simulated peak positions. These were then classified as ‘correctly identified’ peaks. As can be seen the probability of missing Bragg peaks with PF8 increases as the percentage of outliers increases (*i.e.* at higher Bragg peak densities). However, this also depends on how the values of outliers are distributed. If there are a large number of Bragg peaks with intensity values close to the cut-off threshold then the probability that PF8 will miss the weaker peaks increases. This most often occurs within the higher-resolution shells where the Bragg peak intensities are normally weakest with an SNR only just above the background.

**Figure 3:**
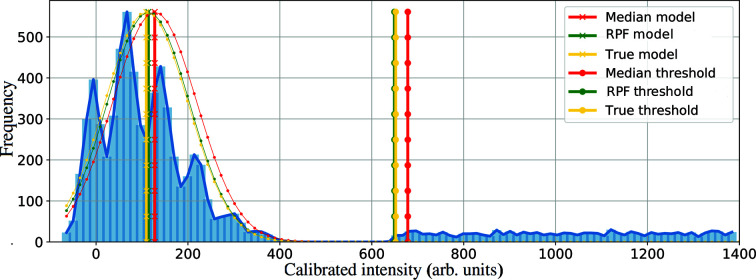
Histogram of pixel intensities with added synthetic Bragg peaks. The true model of the data and cut-off threshold (yellow) is shown along with median estimation and median cut-off (red) and the proposed RPF model estimation and the proposed RPF cut-off threshold (green). In this simulation the median misses some of the Bragg peaks which are located on the left hand side of the threshold (red) owing to the presence of many outliers.

The improvement made to the model parameter estimation using established robust statistics (*e.g.* the median model) can also be tested using a similar approach to the one above. As can be seen in Fig. 3 ▸, the median model, when there is a high density of Bragg peaks in a particular region, results in a less accurate estimate of the true average of the background density and results in some Bragg peaks being missed. Even though the median approach is a robust estimate based on the inliers, we observe that increasing the number of outliers still affects the median value. An extreme scenario is when 49% of data are outliers. In such a case the median is calculated using inliers that are the furthest from the true average.

**Figure 4:**
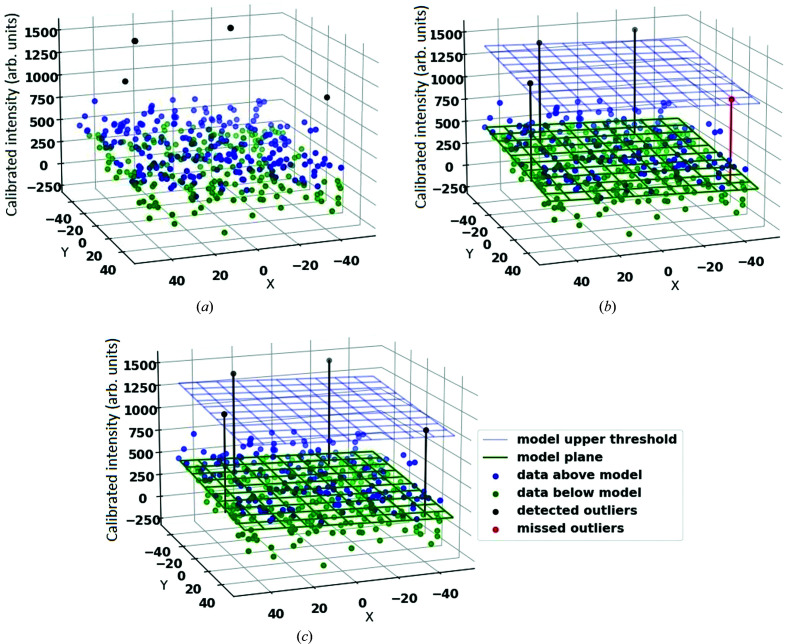
An example of geometric model fitting for a noisy data set including 400 inliers representing the background intensities and four outliers (Bragg peak intensities) that are arranged in close proximity to one another and close to the acceptable threshold. (*a*) The diffraction data, with the *x*–*y* axes representing the position of pixels on the 2D detector and the abscissa representing the calibrated pixel intensities. (*b*) The background is modelled using a non-robust statistics approach (PF8). This results in the loss of outliers (Bragg peaks) which are highlighted in red. (*c*) Using robust statistics allows for modelling the background without including the outliers. In this case all of the Bragg peaks are detected.

In the current approaches to peak finding mentioned above, the local background intensity of a Bragg peak is modelled with a one-parameter model. However, since the average intensity of pixels is a function of resolution, a model with more degrees of freedom is needed to capture this change in pixel intensities on different resolution rings. In this paper we propose to model the local background intensities with a four-parameter plane that can tilt according to the background gradient. We will describe the details of the plane fitting method in Section 4. To show the effect of using robust methods, we consider the example of fitting a four-parameter plane to the background intensities shown in Fig. 4 ▸. This example uses a simulated noisy data set and contains outliers (Bragg peaks) having values close to the tail of the noise distribution. Fig. 4 ▸ shows the results of using both robust and non-robust methods and demonstrates that robust methods are more reliable for detecting subtle differences between inliers and outliers.

**Figure 5:**
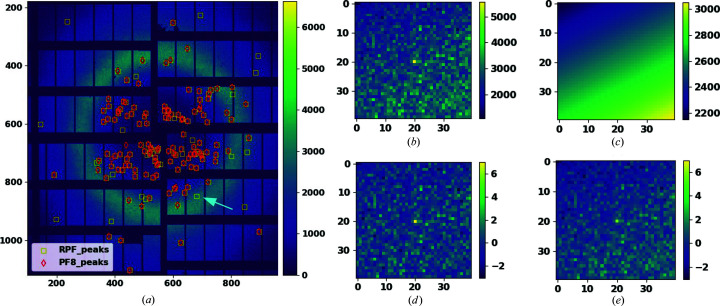
Analysis of a representative diffraction image from the EuXFEL data set. (*a*) A diffraction pattern chosen from the EuXFEL data set with peaks identified using RPF (yellow markers) and PF8 (red markers). (*b*) A Bragg peak and its local background detected with RPF and missed by PF8. (*c*) The local background intensities estimated with a tilted four-parameter plane using the RPF method. (*d*) SNR for a single Bragg peak isolated from the image in (*a*), as indicated by the arrow, estimated using a robust method (RPF) and (*e*) SNR for the same Bragg peak isolated in (*d*) but estimated using the non-robust method (PF8).

Fig. 5 ▸ presents an example of a diffraction pattern taken at the EuXFEL along with the Bragg peaks found by both the RPF and PF8 methods. RPF was able to detect more Bragg peaks than the PF8 program in this pattern. Fig. 5 ▸(*b*) shows an example of Bragg peaks detected with RPF and missed using PF8. Fig. 5 ▸(*c*) illustrates the local background intensities estimated with a tilted four-parameter plane using robust methods (RPF). Figs. 5 ▸(*d*) and 5 ▸ ▸(*e*) show the estimated SNR for each pixel surrounding the same Bragg peak using a robust and a non-robust method, respectively. These two figures show that the estimated SNR for the Bragg peak is 6.3 using the robust method and 5.8 using the non-robust method.

## 4. Methodology   

### 4.1. Robust model fitting   

In order to treat the background using robust statistics, the background noise is modelled using a Gaussian probability density function (PDF) in the presence of outliers that are independent and identically uniformly distributed.

We make use of two methods in statistical analysis: (i) fast least *k*th order statistics (FLkOS) (Bab-Hadiashar & Hoseinnezhad, 2008 ▸), an optimization method that finds the best fit for the model (for example as shown by the green plane in Fig. 4 ▸), and (ii) modified selective statistical estimator (MSSE) (Bab-Hadiashar & Suter, 1999 ▸), a noise scale estimator that gives the standard deviation of the Gaussian model. The model fitted using FLkOS and the scale estimated by MSSE are used to define the threshold to separate outliers from inliers [for example as shown by blue plane in Fig. 4 ▸(*c*)]. These methods are described briefly below.

#### 4.1.1. Fast least *k*th order statistics   

FLkOS is an optimization method that minimizes the *L*_∞_ norm (the largest value of any set of scalar values) of model fitting errors of the inliers (Bab-Hadiashar & Hoseinnezhad, 2008 ▸). The method takes the minimum number of inliers as the input, denoted by *k*, and finds the best parameters for the model. With respect to peak finding for serial crystallography, as mentioned previously, the background pixels are classified as inliers (B) whilst the Bragg peak pixels are classified as outliers (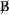). In this case, the goal is to robustly fit a plane to the background data.

Given that only a portion of the data can be used to robustly find the parameters of the model, the optimization method is designed to seek an optimum subset of data. Among all possible subsets of *X* denoted by *e* here (*e* ∈ *X*), some may have lower fitting errors according to a particular cost function. The least *k*th order statistics (LkOS) cost function (Tennakoon *et al.*, 2016 ▸) is used for the model fitting to the subset of data *e*. Given a subset *e*, the parameters of the model θ_*e*_ are obtained by fitting the model to data points in *e* according to the linear regression method (Huber, 2009 ▸). The linear regression method minimizes 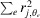, where 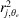 is the squared algebraic distance of the *j*th data point in *e* from the model with parameters θ_*e*_. Afterwards, the squared fitting errors, denoted by 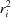 for the *i*th pixel, are calculated for all data points in *X* with respect to θ_*e*_. The pixels in *X* are sorted according to their errors in ascending order by indices denoted by *I*, *i.e.* {*r*_*i*_} (*r*_*i*_ ≤ *r*_*j*_ if *I*_*i*_ ≤ *I*_*j*_). The LkOS cost function is defined as 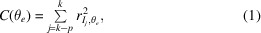
where 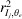 is the *j*th sorted squared fitting error with respect to the model with parameters θ_*e*_. This cost function sums the squares of fitting errors of *p* data points which, after sorting, are ordered by *k* − *p* to *k* indices. The values of *p* and *k* are fixed and pre-defined as discussed shortly.

To seek the optimum subset of data, the FLkOS optimization algorithm is incorporated to minimize this cost function. The optimization is initialized with a set of model parameters θ_*e*_. The squared fitting errors, 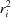 are calculated for all data points with respect to θ_*e*_ and sorted in ascending order (sorting indices are denoted by *I*). The strategy of FLkOS embeds the calculation of derivatives of the cost function in sampling a new subset from sorted residuals, 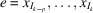, which is the set of furthest *p* inliers to the current model. Subsequently, the model parameters are updated by linear regression carried out on the new sampled subset. FLkOS runs these steps iteratively until convergence of the cost function *C*(θ_*e*_) [equation (1)] or until a pre-defined threshold is reached after a set number of iterations (Bab-Hadiashar & Hoseinnezhad, 2008 ▸). Here, *p* is the sample size, which we have taken to be ρ + 4 [adapted from the article by Purkait *et al.* (2017 ▸)], where ρ is the number of parameters of the model (in the case of fitting a scalar value or a horizontal plane, ρ = 1, and in the case of fitting a tilted plane, ρ = 4).

The success of the above optimization algorithm depends on the input parameter *k* (Sadri *et al.*, 2018 ▸). It should be below the possible number of outliers in any window around Bragg peaks. In the case of crystallography, we assume that at least half of the data points belong to inliers. *k* has a lower bound as it cannot be less than the number of parameters in the model. However, a larger *k* allows for a more accurate linear regression (Hoseinnezhad *et al.*, 2010 ▸). We conservatively assume a value of *k* = 0.5*N* here, where *N* is the number of data points in *X*. Since half of the closest set of residuals are all inliers, this ensures the convergence of the algorithm. As such, the RPF method will function at least as well as the median method which can start to be inaccurate as the number of outliers increases.

#### 4.1.2. Modified selective statistical estimator   

Given the final optimized model parameters, MSSE (Bab-Hadiashar & Suter, 1999 ▸) is an approach often used for separating outliers from inliers. First, the fitting errors of all data points, 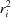, are calculated and sorted (denoted by 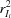 after sorting). The MSSE method then finds the final set of all inliers. After sorting, all data points ordered after the 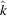
th data point are outliers if 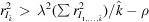. In other words, the outliers have fitting errors that are larger than λ times the standard deviation of the inliers. The parameter λ is taken to be between 2 and 4 in the statistics literature (Huber, 2009 ▸). This is based on the fact that 95 to 99.9% of a Gaussian probability density distribution lies within 2 to 4 times its scale. The sensitivity of the proposed method to this parameter is further discussed in Section 5.4. This allows segmentation of inliers according to their density, regardless of the density of outliers, which is one of the improvements of the RPF approach over PF8.

#### 4.1.3. Peak finding   

Peak finding involves the analysis of data points which comprise the pixel intensity values of the detector. The goal is to model the local background intensities of a Bragg peak by fitting a plane. The number of pixels comprising the local background is fixed and defined as will be discussed in this section. The algorithms in PF8 and our previous work (Hadian-Jazi *et al.*, 2017 ▸) model the background using a single-parameter horizontal plane, assuming a constant background intensity. This results in an inaccurate estimate of the background mean, where the image has a noticeable non-zero gradient. For example, this is most apparent in regions closest to the water ring, where the background has a strong gradient. The RPF method described here improves on this by providing the possibility of fitting a tilted plane to the background independently of the presence of outliers (Bragg peaks), *i.e.* following the principles of robust statistics.

A further improvement of the RPF method is that in the presence of a large number of outliers (*e.g.* Bragg peaks or particularly noisy pixels) it is possible that the median is far from the mode of the noise distribution, as can be seen in Fig. 3 ▸ (the median is only considered to be a robust statistic when a significant majority of the data points are inliers). Statistics such as the median are less useful when a greater number of outliers are present, as the median in turn becomes more separated from the mode of the probability density function. The robust statistics approach proposed here is not affected by the number of outliers.

After estimating θ_*e*_ (four parameters of the plane), the estimated σ_*e*_ is used to define the SNR for a given pixel *x*_*i*_. In our method, similar to PF8, pixels with SNR above a given minimum acceptable threshold are assumed to be Bragg peaks.

### 4.2. Robust peak finder   

**Figure 6:**
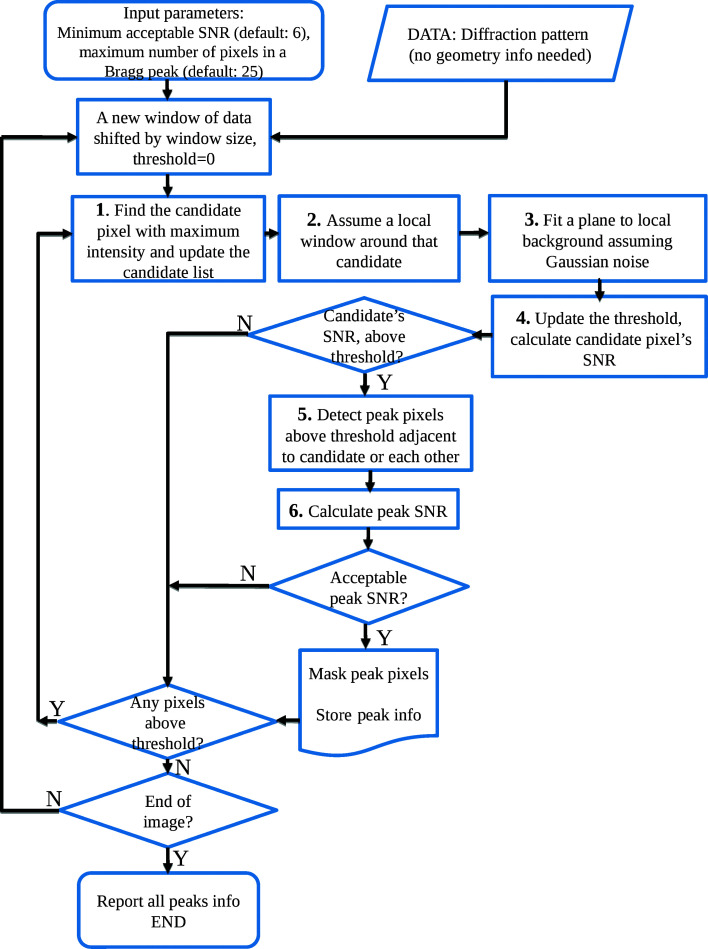
Flow chart illustrating the steps of the RPF algorithm.

Fig. 6 ▸ is a flow chart of the RPF algorithm. Briefly, the algorithm proceeds as follows: First, the algorithm takes as its input the diffraction pattern or a region of it; for the RPF approach the geometry of the detector (in terms of relative position of panels with respect to one another or to the beam, termed the ‘geometry file’) does not have any influence on the results. This is an important difference between the RPF and PF8 algorithms and is possible because modelling of the background is performed locally within a window around candidate Bragg peaks using a tilted plane. Two main input parameters, the minimum acceptable SNR and the maximum number of pixels of a Bragg peak, are required. The latter is used to define the size of the window around candidate peaks in order to model the background.

Using a shifting window over the whole image with a step size of one window width, the algorithm starts searching for candidate peaks at the corner of the image. Initially, the threshold for the background intensity is zero. At each position of this shifting window, it finds a pixel that (*a*) has not been analysed before, (*b*) is above the background threshold for this window and (*c*) is a local maximum with respect to all other pixels contained within the window. The background of local pixels surrounding this candidate peak is modelled by fitting a tilted plane with four parameters θ_B_ using FLkOS and a noise scale σ_B_ using MSSE. The threshold *T* = μ_B_ + τσ_B_ which separates outliers (candidate Bragg peak) from inliers (the background pixels) is then calculated. If the intensity of the candidate pixel is above this threshold, all the pixels which are adjacent to it and are also above the threshold will be classified as belonging to the Bragg peak. After the peak pixels have been assigned, the SNR for the peak is calculated using 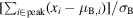, where *x*_*i*_ are the values of pixels belonging to the Bragg peak. If the peak SNR is above the minimum acceptable SNR (τ), the peak information is stored in the output peak list.

After a candidate peak has been analysed, the candidate pixels that have been visited before are flagged and the threshold of the background for the current window is updated to *T*. The algorithm searches for more Bragg peaks by looking for the next Bragg peak candidate. If there are no more candidate peaks, the window is shifted across the image with a step size equal to the window’s width. In each new window, initially no pixel is flagged as no pixel is yet visited and the threshold *T* is set to zero. The above procedure is repeated until there are no more windows for analysis.

### 4.3. RPF implementation   

Many problems in the data analysis pipeline can be reduced to outlier detection when the inliers are modelled by a Gaussian probability density function. For the present work we have developed a software library called the robust Gaussian fitting library (*RGFlib*), based on the above methods. This software library forms the basis for the RPF algorithm in this paper. Issued under the GNU licence, the developed library is publicly available for the wider community (Sadri & Hadian-Jazi, 2020*b* ▸) and applications.

We have implemented the RPF method in two different software packages which use the *RGFlib* library. The first is a standalone version of RPF. To use the RPF standalone implementation, we have added a Python wrapper and a set of scripts that are accessible to general users. This implementation can be found in the publicly available Git repository (Sadri & Hadian-Jazi, 2020*a* ▸). All basic functions in *RGFlib* come in two forms, serial or parallel processing, using the built-in multiprocessing available in the Python programming language. This will dramatically speed up data reduction when using computing clusters. The standalone implementation of RPF requires the input to be in HDF5 format. Existing programs such as *cdf2hdf5* can be used to convert other file formats to HDF5.

The second program incorporates the RPF method into *CrystFEL*, one of the most commonly used software packages for performing SFX data analysis. In order to use this extension, in *CrystFEL*, the option --peakf inder = robustpeakfi nder must be selected (https://gitlab.desy.de/alireza.sadri/crystfel).

#### 4.3.1. Input parameters   

The RPF input parameters include the minimum and maximum number of pixels in a peak (the default parameters are set to 1 and 25, respectively) and the minimum acceptable SNR (the default is 6.0). The maximum threshold for the background mean (default is +∞), the maximum number of Bragg peaks in a frame (the default is 1024) and the bad pixel mask are additional inputs for the program. One important input is the threshold for the dark field, which must be set to the standard deviation of the detector pixels without any photons incident upon the detector. This input is used to determine reliable values for the background model parameters as described in Section 5.5. The parameters of the algorithm are easy to tune and in practice we have found that the only parameter that may benefit from tuning is the SNR threshold value, which we recommend is set to 6.0 for data reduction; this is the value used for the tests in Section 5. It is possible to also set the resolution limits for the peak finder but this step is not essential. The RPF program can be configured to return a mask showing the positions of detected Bragg peaks.

PF8 requires additional information regarding the geometry of the detector in order to accurately position each of the modules with respect to one another. Consequently, the algorithm is unable to analyse data from the individual modules in parallel. Our modification of PF8 incorporating the RPF algorithm currently does not remove this limitation.

Generally, to detect weak Bragg peaks in diffraction images, it is common practice to reduce and optimize the minimum acceptable SNR threshold, τ. During our tests, we did not observe any significant benefit to reducing the SNR threshold value in terms of the overall accuracy. Therefore, we recommend using the default SNR threshold value. In Section 5.4, we discuss the sensitivity of RPF to the changes in the SNR in more detail.

#### 4.3.2. Scalability   

The usual method for parallelization of SX data analysis is by running a peak finder over a set of complete diffraction patterns using multiple processors in parallel. The offline software program that we have prepared for the use of the RPF method can analyse a stack of images from a single module and report peak lists for each module individually. This is particularly useful for fast detectors such as AGIPD (Allahgholi *et al.*, 2019 ▸), which saves image data module wise.

An online monitoring software can potentially avoid transferring the raw data from modules into memory if the frame is not a hit, but using radial information imposes a limitation on such approaches. To analyse the intensity of pixels on the same radius from the centre of the image, the large set of images from all modules must be loaded into a single memory in one computational node. This requires a huge amount of memory, which is very expensive, and the transmission is time consuming. However, the advantage of RPF is that, by using hardware such as field programmable gate array (FPGAs) or GPUs directly connected to a detector, analysing each module independently is possible and can potentially make the process very fast. In this scenario, a processing core could be assigned to each module and only when the total number of Bragg peaks over all modules is above a given threshold (a hit) is the set of images transmitted over the network into storage units.

For peak-finding methods using radial information and for detectors with multiple separate modules, inclusion of additional detector geometry information about the relative position of modules with respect to each other and to the beam is necessary. Such methods are sensitive to the accuracy of the estimated position and orientation of modules. During offline processing, this sensitivity is dealt with by refining the detector’s geometry description using the data collected after the experiment. This refinement can be challenging for online monitoring. RPF does not require any radial information, which allows analysis of modules individually in parallel. This scalability is a promising feature for RPF, particularly for detectors with a large number of modules.

## 5. Proof of concept (algorithm testing)   

In this section, we present an evaluation of the performance of the RPF algorithm on a selection of data sets: (1) CXIDB entry 32, (2) EuXFEL commissioning test and (3) Petra III p11 data set. Table 1  ▸ provides an overview of the three data sets including the data collection parameters and their unit cells. In summary, these data sets were specifically chosen to test (i) the sensitivity in identifying peaks which are located very close to one another (CXIDB 32 data set), (ii) the compatibility of the RPF algorithm with the AGIPD detector (EuXFEL commissioning data set) and (iii) its accuracy in identification of weak Bragg peaks having low SNR (Petra III p11 data set).

We used an analysis pipeline that comprised multiple stages. The first step was to correct the data according to the calibration constants of the detector. Afterwards, the calibrated data were passed to the peak finder to reduce the data set to only useful frames (*i.e.* those containing crystal hits) and generate data sets that included data and metadata only for these hits. We evaluated the peak-finding method in *Cheetah* (PF8) (Barty *et al.*, 2014 ▸) and the RPF approach. To confirm that the RPF algorithm identified ‘true’ Bragg peaks and not random peaks that may be present in the background, the RPF peak list was run through the standard crystallography indexing programs that are incorporated within *CrystFEL *(White *et al.*, 2012 ▸). The difference in the number of peaks identified before and after indexing provides an indication of the level of accuracy of the peak-finding algorithms. An important consideration is the fraction of ‘noisy’ pixels that the RPF and PF8 algorithms incorrectly assign as ‘real’ peaks. One way to compare the respective performance of the two algorithms for distinguishing actual Bragg peaks from noise is to look at the indexing rates for the two algorithms using a common data set. Table 2 ▸ summarizes the results for the three data sets. In the case of the Petra III data set, RPF assigned 55 748 hits, which resulted in 26 346 frames being indexed (47.26%). For the same data set the PF8 algorithm assigned a much larger number of hits (453 231). However, only 23 864 were indexed (5.26%), indicating that a much larger proportion of ‘hits’ are actually just noise when using the PF8 algorithm. Therefore, the much higher indexing fraction achieved using the RPF versus PF8 algorithm indicates that the former is more robust with respect to noisy data containing weak Bragg peaks.

*CrystFEL* version 0.9.1 is used in our analysis. At this point the self-consistency statistics 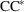, *R*_split_ and CC_1/2_ were generated, and these results are reported and compared for each analysis test. The overall figures of merit are discussed, and we also provide figures for the high-resolution data. These three parameters are figures of merit in crystallography and indicators of data quality. They are defined as follows: CC_1/2_ is a linear correlation coefficient between intensity estimates from half data sets and is helpful in determining the resolution cut-off for the data set (Karplus & Diederichs, 2015 ▸). 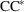 provides a cross-validation-independent indication of overfitting and is calculated as 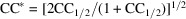 (Karplus & Diederichs, 2015 ▸). *R*_split_ or the self-consistency *R* factor is an unweighted sum of intensities for merged data (Karplus & Diederichs, 2015 ▸). *R*_split_ is equivalent to *R*_pim_, which is an adaptation of the *R*_merged_ for conventional crystallography data collection.

In order to directly compare each data set, the raw data were treated identically in each case. The same bad pixel mask was used for both the RPF and PF8 peak finders. The previously published bad pixel mask was used for the CXIDB32 and Petra III data sets. For the EuXFEL data set a recently developed bad pixel mask algorithm was used (Sadri *et al.*, 2021 ▸). By applying an identical bad pixel mask, irrespective of the specific hit-finding algorithm used (RPF or PF8), any bias due to the application of the mask was avoided. The bad pixel masks for each detector are designed to include a border at the edge of the detector panels to mask out spurious pixels within this region. The input parameters for indexing/merging were also fixed as this allows us to study the effect of changing the peak-finding method only.

Whilst the RPF approach is able to achieve reasonable peak-finding results as a standalone program, one of the main purposes of developing this method is online data reduction. Our solution to data reduction is to either ignore or delete frames of data which do not contain any Bragg diffraction data by applying the RPF approach. In contrast to PF8, the goal of RPF is to adopt a largely unsupervised approach to rapidly determine whether or not a given detector frame contains data. To verify that RPF can be used for data reduction, we compared the performance of PF8 (with optimized parameters set) before and after data reduction by RPF. Three analysis tests were conducted; their results are presented here for each data set. The tests can be summarized as follows:

(1) Run PF8 to obtain results.

(2) Run RPF to obtain results.

(3) Run RPF for initial hit finding followed by running PF8 on the hits stored by RPF. This allows further offline optimization to see if we can achieve better results.

We performed the above tasks on the CXIDB32, EuXFEL commissioning and Petra III p11 data sets. The size of the shifting window for the AGIPD, PILATUS and CSPAD detectors was set to 16 × 16, 32 × 32 and 32 × 32 pixels, respectively, based on the number of pixels defined per Bragg peak for the individual data sets. The size of the shifting window is adjusted depending on the expected maximum size of a Bragg peak and their relative distance from one another (determined by the size of the unit cell) and is limited to ∼5 Bragg peaks per window area. The default value for this parameter in RPF is set to 16 × 16, based on the fact that the default value for the maximum number of pixels in a given peak is set to 25 pixels.

**Figure 7:**
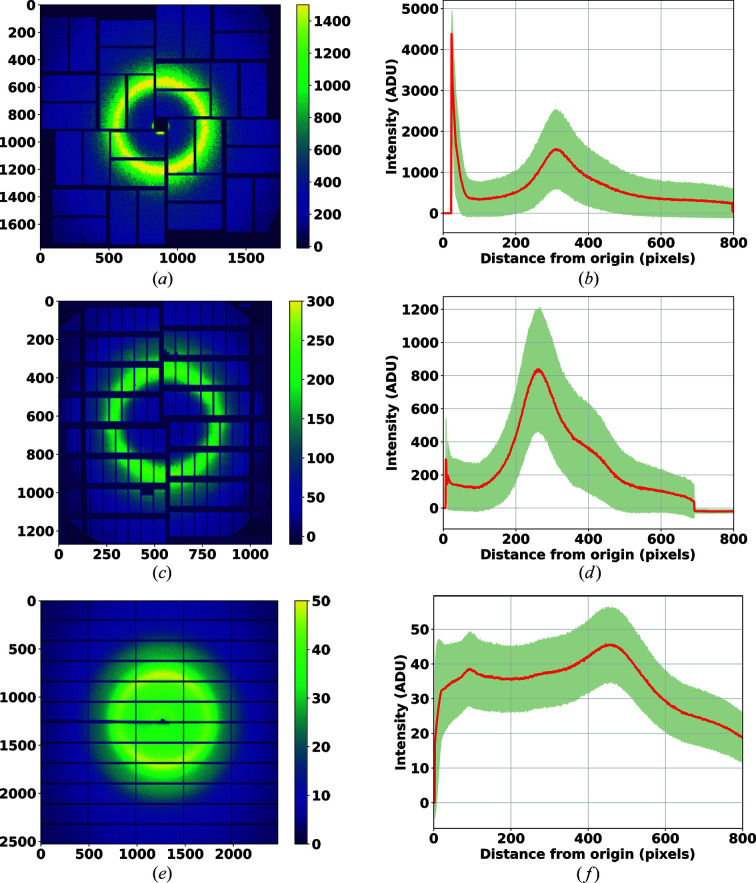
Background analysis for three tested data sets. Temporal average diffraction pattern and radial averaging of the background for data set (*a*), (*b*) CXIDB 32 (*c*), (*d*) EuXFEL commissioning and (*e*), (*f*) Petra III. These figures are generated with diffraction patterns with Bragg peaks omitted.

In order to provide further insight into whether RPF is able to more accurately identify Bragg reflections in the three data sets tested, we analysed the level of background fluctuation in the images (Fig. 7 ▸). Figs. 7 ▸(*a*), 7 ▸(*c*) and 7 ▸(*e*) present the temporal average of diffraction patterns for each of the three data sets, CXIDB32, EuXFEL commissioning and Petra III, respectively. These figures were generated by averaging all of the diffraction patterns associated with each data set with the Bragg peaks excluded. Figs. 7 ▸(*b*), 7 ▸(*d*) and 7 ▸(*f*) shows a 1D plot generated from the radial-background images in Figs. 7 ▸(*a*), 7 ▸(*c*) and 7 ▸(*e*), respectively. The shaded areas in these figures represent three standard deviations of the intensity from the radial average of the background. The EuXFEL data set shows a very high level of background variation (±500) compared with other two data sets, while Petra III had the lowest background variation between images (±10), in spite of having an overall high background signal due to Kapton. The peaks in Figs. 7 ▸(*b*), 7 ▸(*d*) and 7 ▸(*f*) show the solvent ring present in the data. Table 2 ▸ summarizes the results for the three data sets.

### 5.1. CXIDB32 data set   

In this section we present the results of applying the RPF algorithm to the data set of Zhou *et al.* (2016 ▸). This data set was collected at the LCLS CXI beamline, on the rhodopsin–arrestin complex. The detector used was the CSPAD (Herrmann *et al.*, 2013 ▸). The raw data are publicly available and accessible via the CXI Data Bank (Maia, 2012 ▸) (CXIDB32; https://doi.org/10.11577/1241101). The data set was chosen because it has a relatively large unit cell, resulting in closely spaced Bragg peaks, and a low-angle lipid cubic phase (LCP) background scatter. These characteristics make the data set challenging for peak-finding algorithms. Therefore, this data set was chosen to help assess the reliability of our RPF algorithm in correctly identifying peaks. We compared the RPF results with the PF8 peak-finding results. Zhou *et al.* (2016 ▸) analysed the structure by sorting the data into three batches. Of these batches, two were deemed of sufficient quality for structural analysis. For this analysis the acceptable SNR for RPF was left at the default value (τ = 6); for PF8 an SNR threshold value of six was chosen to match that used in the published results. In this experiment, the minimum number of Bragg peaks in a diffraction pattern classified as a hit was set to 35. Indexing was performed using the *indexamajig* program – part of the *CrystFEL* package. The parameters used were based on the relevant published indexed data parameters (Zhou *et al.*, 2016 ▸). Briefly, the following parameters were set: the indexing used mosf lm-cell-nolatt, mosf lm-latt-nocell, dirax, asdf, xds-cell-latt, xgandalf and -tolerance= 5,5,5,1.5 -int-radius=2,2,3. The *partialator* command within *CrystFEL* was used for merging the data with the following parameters: -y mmm, -no-logs, -iterations=1, -model=unity, -max-adu=14000, -min-measurements=3. These parameters were kept fixed in order to test the PF8 and RPF results.

Out of a total of 4 046 425 data frames, PF8 detected 22 462 frames which were classified as hits. This gives an overall hit fraction of 0.55%, which is identical to what has been reported (Zhou *et al.*, 2016 ▸). The RPF algorithm detected 58 695 frames which resulted in an increase in the total hit fraction to 1.45%. After application of the peak-finding algorithms, the hits were indexed using *CrystFEL* (White *et al.*, 2012 ▸). The number of indexed frames for PF8 and RPF were 21 875 and 54 359, respectively (with an indexing fraction of 97.39 and 92.61%). For the third test PF8 was run on the hits found by RPF (58 695 frames) and the results indexed (using the new peak lists generated by PF8). *CrystFEL* indexed 36 369 frames from the PF8 peak lists, resulting in an indexing fraction of 61.96% [using the same indexing routines and parameters as Zhou *et al.* (2016 ▸)]. Table 2 ▸ summarizes the results for this data set. This means that if RPF was initially used for data reduction and the results used as an input for PF8 with optimized parameters, PF8 would achieve similar results to those obtained assuming the data had not been reduced. In this analysis, the number of frames indexed by PF8 using the raw data (21 875 indexed frames) was less than when the RPF algorithm was used for the initial hit finding followed by application of PF8 for refinement of peak detection (36 369 indexed frames). In other words, PF8 found fewer peaks in the patterns so there were fewer patterns for the indexer to use to find indexing solutions. We conclude that RPF is able to generate a more complete list of indexable patterns than PF8, and RPF can reliably retain useful crystallographic data whilst achieving a significant level of data reduction.

**Figure 8:**
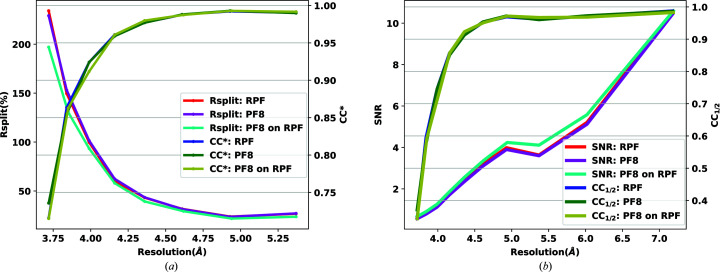
Comparison of the (*a*) *R*_split_ and 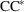 and (*b*) SNR and CC_1/2_ values as a function of resolution (Å) for the CXIDB32 data set. Three tests were performed comparing RPF, PF8 and RPF + PF8 peak-finding algorithms.

Fig. 8 ▸(*a*) shows a comparison of 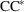 and *R*_split_ for the three test cases and Fig. 8 ▸(*b*) presents the comparison of CC_1/2_ and SNR.

The results show that the RPF algorithm is able to detect more hits from the raw data set which can also be indexed and thus increases the indexing fraction.

### 5.2. EuXFEL commissioning data set   

In this section we present the results of testing the RPF algorithm using an EuXFEL commissioning data set generated from lysozyme crystals (Kirkwood *et al.*, 2021 ▸). The data set was collected at the SPB/SFX instrument (Mancuso *et al.*, 2019 ▸) in March 2020. The beam was delivered with a mean photon energy of 9.3 K eV, 1.1 MHz repetition rate pulses and 352 pulses per train. The AGIPD-1M detector (Allahgholi *et al.*, 2019 ▸) was used and located about 129 mm downstream of the sample. The EuXFEL lysozyme commissioning data set was used as a model system to test if RPF is suitable for online data reduction at the SPB/SFX beamline using the AGIPD detector. The data set includes a number of runs with different settings. We focused on three specific runs (95, 96 and 97) which contain ∼5.7 million diffraction patterns.

For this analysis the threshold SNR was set to the default value of τ = 6 for both the RPF and PF8 algorithms. The minimum number of Bragg peaks in a diffraction pattern to be identified as a hit was set to 20. Indexing was performed using the *indexamajig* program – part of the *CrystFEL* package. The following parameters were used: -int-radius=2,4,7 using the default indexing methods (mosf lm-cell-nolatt, mosf lm-latt-nocell, dirax, asdf, xds-cell-latt, xgandalf). The *partialator* program was used within CrystFEL to merge the data with the following parameters: -y 4/mmm, -min-res=3, -push-res=1.0, -no-logs, -iterations=3, -model=unity. These parameters were kept fixed in order to test the PF8 and RPF results.

**Figure 9:**
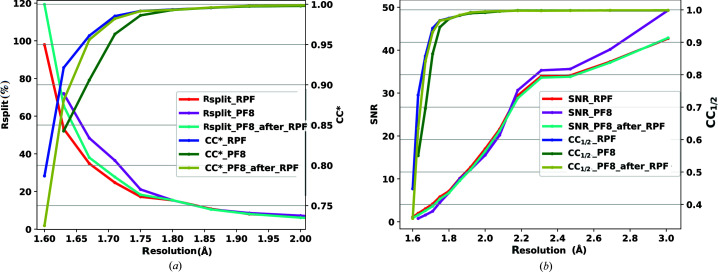
Comparison of the (*a*) *R*_split_ and 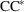 and (*b*) SNR and CC_1/2_ values as a function of resolution (Å) for the EuXFEL lysozyme commissioning data set. Three tests were performed comparing RPF, PF8 and RPF + PF8 peak-finding algorithms.

Of the 5 645 342 frames collected in the three runs, PF8 classified 3 422 532 frames as hits, giving a hit fraction of 60.63%, whilst the RPF algorithm detected 2 127 935 frames, giving a hit fraction of 37.69%. The indexing fraction for PF8 was 36.73% and for RPF it was 81.90% (1 257 048 and 1 742 777 indexed frames, respectively). The output of RPF was run again through PF8 and, from 2 127 935 hits found by RPF, 1 663 851 frames were indexed with *CrystFEL* (using the PF8 peak lists) with an indexing fraction of 78.19%. Table 2 ▸ summarizes these results. One key point to consider from the statistics is that, in this data set, data reduction using the RPF algorithm was found to be more effective and accurate in reducing the data set. Although PF8 found more hits than RPF these were not all indexed and did not end up being used in the analysis. The RPF algorithm resulted in more indexed patterns than PF8, which is a key metric. The final results of RPF (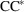 and *R*_split_) are very similar to although slightly better than those for PF8, indicating that RPF has not lost any useful information during the data reduction process, as indicated in Fig. 9 ▸.

### 5.3. Petra III p11 data set   

The third data set tested was collected at the Petra III p11 beamline (Burkhardt *et al.*, 2016 ▸) on dioxygenase using a 12 keV incident photon energy. The detector used for this experiment was a PILATUS 6M (Broennimann *et al.*, 2006 ▸), which was located approximately 250 mm downstream of the sample. More information on the experimental setup is given by Beyerlein *et al.* (2017 ▸). However, the dioxygenase data set is unpublished. The unit cell and PF8 optimization parameters were sourced from Oberthuer *et al.* (2016 ▸). The raw data set is in the CBF format, which is supported in the latest version of *CrystFEL* v9.1 and is automatically converted to HDF5 for hit finding. The dioxygenase Kapton tape drive data set was chosen to test the reliability of the peak-finding algorithms. This data set proved to be challenging to analyse for PF8 (Oberthuer *et al.*, 2016 ▸). The optimal PF8 parameters for this data set had an SNR set to 4 in order to identify all of the Bragg peak positions in the data, which is comparatively low. However, increasing the SNR threshold above this did not provide adequate results in terms of the number of Bragg peaks identified. An SNR threshold of 4 resulted in a 100% hit rate using PF8 with no images excluded from the data set. Owing to the poor SNR, this data set is ideal to test the accuracy and sensitivity of the RPF algorithm in correctly assigning weakly diffracting Bragg peaks. For this analysis the acceptable SNR for the RPF algorithm was left at the default value (τ = 6) and for PF8 it was set to four, as the Bragg peaks were very weak in this data set. In this experiment, the minimum number of Bragg peaks required to be detected in a diffraction pattern in order to be identified as a hit was set to five for both programs. Indexing was performed by *indexamajig* within the *CrystFEL* package. The following parameters were used based on published results (Beyerlein *et al.*, 2017 ▸): for indexing methods mosf lm-cell-nolatt, mosf lm-latt-nocell, dirax, asdf, xds-cell-latt and xgandalf were chosen with -int-radius=2,3,4. The *partialator* program was used within *CrystFEL* to merge the data with the following parameters: -y mmm, -model=unity, -iterations=3, -push-res=1. These parameters were kept fixed in order to test the PF8 and RPF results.

From 453 231 frames collected for this experiment, PF8 detected 453 231 hits, giving a hit fraction of 100% whilst the RPF algorithm detected 55 748 frames, reducing the hit fraction to 12.30%.

**Figure 10:**
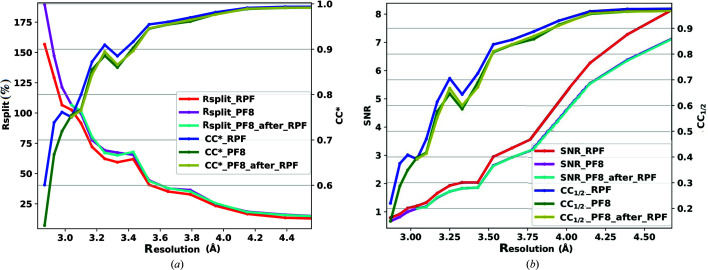
Comparison of the (*a*) *R*_split_ and 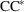 and (*b*) SNR and CC_1/2_ values as a function of resolution (Å) for the Petra III p11 beamline data set. Three tests were performed comparing RPF, PF8 and RPF + PF8 peak-finding algorithms.

The indexing fraction for PF8 was 5.26%, compared with 47.26% for RPF (23 864 and 26 346 indexed frames, respectively). This result shows that whilst the RPF algorithm identified fewer ‘hits’ it found a far higher number of ‘quality’ hits, indicating that the RPF approach is a reliable, robust method for reducing data. We also ran PF8 on the output of the RPF algorithm and indexed the results from the 55 748 hits found by RPF. *CrystFEL* indexed 21 526 frames using the PF8 peak lists, resulting in an increased indexing fraction of 38.61% compared with the original PF8 hit finding but still a reduced indexing fraction compared with the RPF results. Table 2 ▸ summarizes the results of analysing this data set along with the results for the other two data sets. Fig. 10 ▸ presents a comparison of 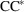, *R*_split_, CC_1/2_ and SNR for this analysis, indicating similar trends for both algorithms. The accuracy of RPF in detecting peaks within regions of high background noise (such as within the solvent ring) is a result of how the local background is modelled using a four-parameter fitting of a tilt plane. This represents a significant advantage for crystallographic data, for example, collected in strongly scattering delivery media (Fig. 4 ▸).

**Figure 11:**
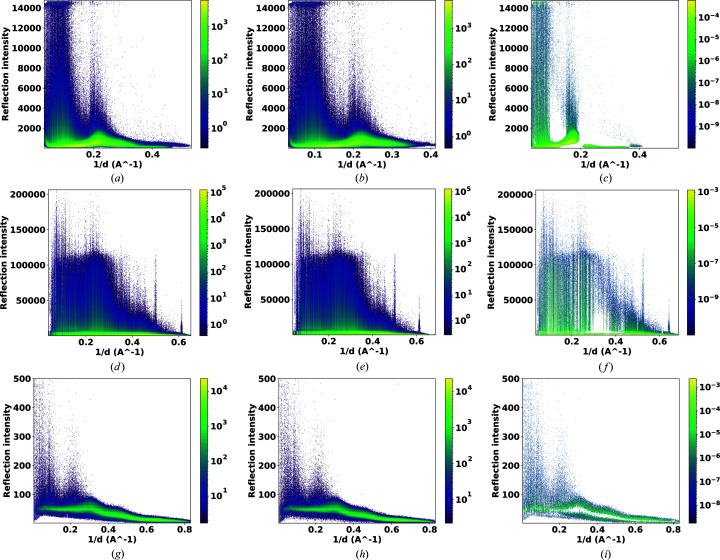
Peakogram histograms showing the highest pixel value for each reflection versus resolution for (*a*) the CXIDB 32 data set, RPF results, (*b*) the CXIDB 32 data set, PF8 results, (*c*) the difference of normalized histograms of (*a*) and (*b*), (*d*) the EuXFEL commissioning data set, RPF results, (*e*) the EuXFEL commissioning data set, PF8 results, (*f*) the difference of normalized histograms of (*d*) and (*e*), (*g*) the Petra III p11 data set, RPF results, (*h*) the Petra III p11 data set, PF8 results, and (*i*) the difference of normalized histograms of (*g*) and (*h*). The histograms were generated using *CrystFEL* *peakogram-stream* (White *et al.*, 2012 ▸).

Fig. 11 ▸ shows the peakogram plots which represents the highest pixel value for each reflection over the resolution range of the data. These plots were generated for the three different data sets using *CrystFEL peakogram-stream* for both the RPF and PF8 methods. Figs. 11 ▸(*a*), 11 ▸(*d*) and 11 ▸(*g*) show the EuXFEL commissioning data set, CXIDB32 data and Petra III data peak-finding results for RPF, while Figs. 11 ▸(*b*), 11 ▸(*e*) and 11 ▸(*h*) are the corresponding results using the PF8 algorithm. The plots identify that the Petra III data set has lower reflection intensities than the other two data sets, confirming the poor SNR in the data, while the EuXFEL data have the highest resolution. However, the intensities and number of peaks for each peak-finding algorithm appear similar. But, if we extract the peaks only detected by RPF and not PF8 [Figs. 11 ▸(*c*), 11 ▸(*f*) and 11 ▸(*i*)], a clear difference between the two algorithms is observed. Figs. 11 ▸(*c*), 11 ▸(*f*) and 11 ▸(*i*) were generated by normalizing the histogram from Figs. 11 ▸(*a*), 11 ▸(*b*), 11 ▸(*d*), 11 ▸(*e*), 11 ▸(*g*) and 11 ▸(*h*), respectively, and then differentiating them. RPF was able to identify more peaks at low resolution in the EuXFEL data set, while in the other two data sets RPF identifies peaks throughout the whole resolution range.

RPF involves a two-stage process of hit finding. The first step performs eight iterations of sorting of pixels within a local area. The computational complexity of this process is linear with respect to the number of pixels in that area. This is because, during sorting, RPF does not sort elements within each partition but rather finds two percentiles of the data and every element in between the two percentiles. The second stage, the scale estimation, involves a full sorting of the elements. These two operations are performed for every candidate Bragg peak. In contrast, PF8 performs five iterations of averaging and calculating the standard deviation over all pixels as a function of radial distance from the centre of the image. This means that the computational complexity of PF8 increases with the size of the image and its speed consequently decreases for detectors with larger pixel numbers.

**Figure 12:**
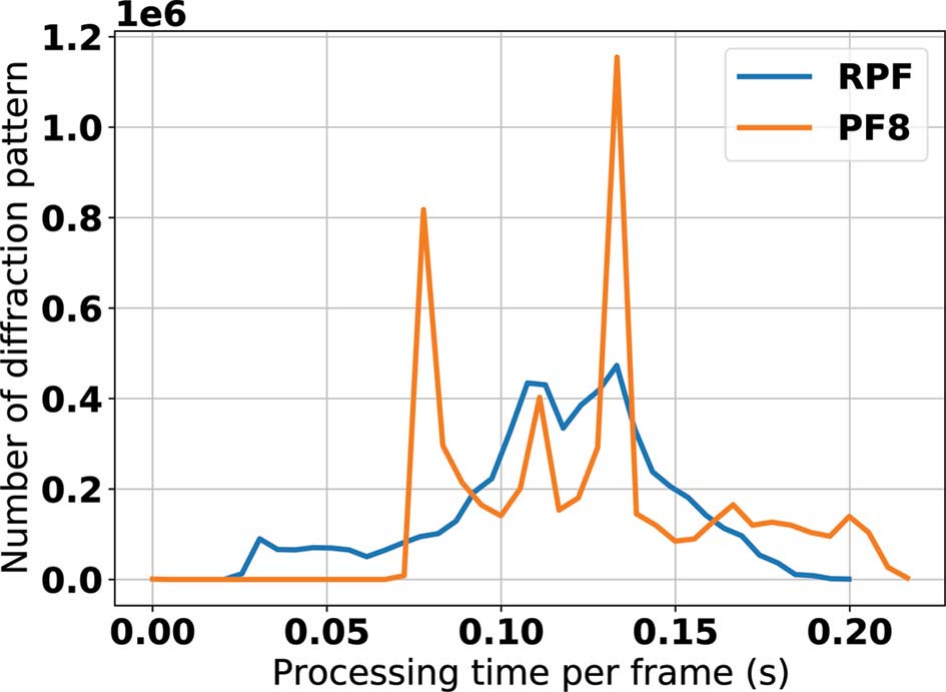
Histogram showing the speed of the two peak-finder algorithms, RPF (blue) and PF8 (orange). Two hundred thousand diffraction images were selected randomly from the EuXFEL commissioning data set and both RPF and PF8 were used to classify images as ‘hits’. The speed with which these algorithms carried out this task is demonstrated by plotting the number of diffraction patterns versus the processing time per frame. The histogram is generated using a single node with 80 cores (INTEL, E5-2698 v4 @ 2.20 GHz, memory 512 GB) from the UPEX partition in the Maxwell computing cluster.

The current implementation of RPF works offline and the results reported here are obtained via offline analysis performed on a high-performance computing cluster (DESY Maxwell). To run the method in ‘real time’ online, a computer (CPU, GPU, FPGA) needs to read the data from each individual module of the detector. To provide an analysis of the potential speed increase that RPF is capable of compared with PF8, a histogram of the number of diffraction patterns versus processing time per frame for 200 000 randomly selected diffraction patterns from the EuXFEL commissioning data set was generated (Fig. 12 ▸). This demonstrates that the RPF algorithm yields a factor of three increase in the hit-finding speed compared with PF8 whilst working offline. Fig. 12 ▸ is generated using a single node with 80 cores (Intel, E5-2698 v4 @ 2.20 GHz, memory 512 GB) from the UPEX partition in the Maxwell computing cluster. The online analysis speed is still to be confirmed, but since the RPF algorithm can be run on multiple detector modules in parallel, the relative difference in speed is expected to be even greater.

### 5.4. Sensitivity analysis   

In order to compare the sensitivity of the two methods with the input parameters, we chose a small subset of the European XFEL commissioning data set, used in Section 5.2, for a sensitivity analysis and varied the input parameters for the two peak-finder methods to observe their behaviour.

**Figure 13:**
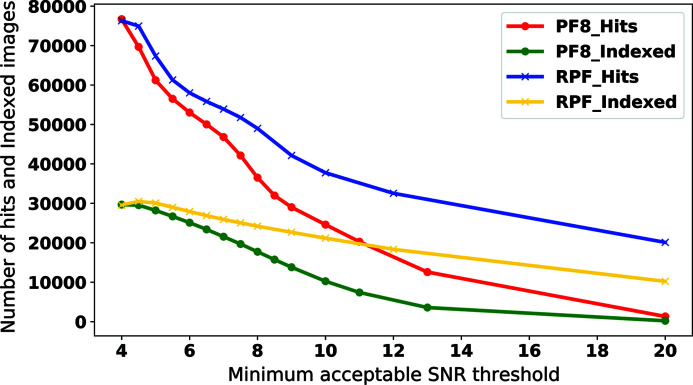
Number of hits and indexable patterns as a function of the minimum acceptable SNR threshold. The number of hits and indexable patterns decrease for both methods with increasing minimum acceptable SNR. In this case, the number of indexable patterns is more stable for RPF than for PF8.

**Figure 14:**
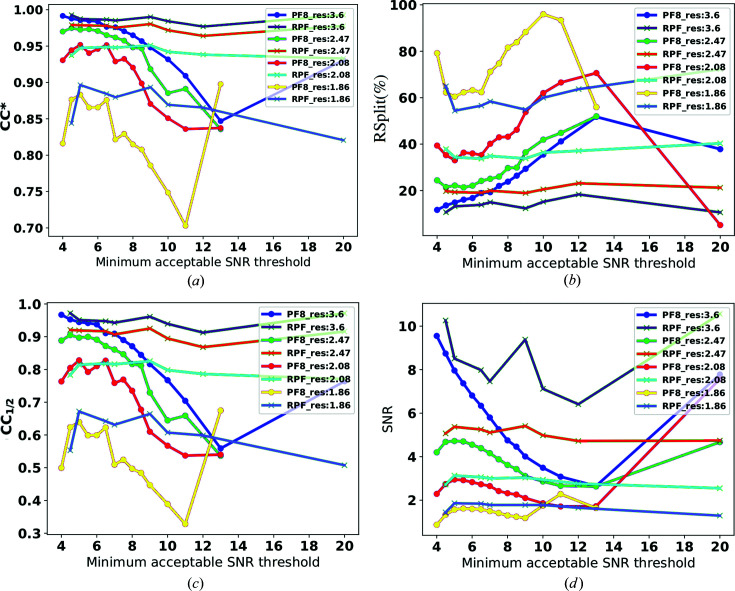
Comparison of the performance of RPF and PF8 methods for (*a*) 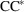, (*b*) *R*_split_, (*c*) CC_1/2_ and (*d*) SNR as a function of the minimum acceptable SNR threshold for four different resolutions. The performance of RPF for all four resolutions in all figures is less sensitive to change in the minimum acceptable SNR threshold. However, the PF8 performance is very sensitive to the change of minimum acceptable SNR threshold.

In this test we varied the minimum acceptable SNR parameter, τ, for the two peak finders and report on the results. The minimum acceptable SNR threshold was chosen assuming no maximum resolution limit. The purpose is to find the regions of τ where performance is optimum for each method and, more importantly, to judge the reliability of the methods and their sensitivity to the input parameters. This is as opposed to comparing the absolute values for performance of the methods as the input parameters are treated differently in these algorithms. This test was performed on a single sequence (number 5) of run 96 of the specified data set. This subset comprises 90 000 X-ray diffraction patterns collected from lysozyme crystals. The results of this study are shown in Figs. 13 ▸ and 14 ▸. In Fig. 13 ▸, the number of hits and indexed patterns with the two methods is shown. Fig. 14 ▸ shows the self-consistency statistics for the evaluation of each method at four different resolutions. The resolutions were randomly selected as 3.6, 2.47, 2.08 and 1.86 Å. The aim of the test is to evaluate the sensitivity of each method with respect to the input parameter. All of the four figures of merit 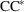, *R*_split_, CC_1/2_ and SNR were stable at all of the resolution points tested for RPF when the input parameter minimum acceptable SNR was varied between 4 and 20. Therefore, the RPF performance was less sensitive to the change in SNR parameter. On the other hand, the PF8 performance was very sensitive to the minimum acceptable SNR parameter, showing a large variation in the four figures of merit when the minimum acceptable SNR parameter was varied between 4 and 20. PF8 is highly tunable, and from this analysis, it seems to give the best results when the minimum acceptable SNR threshold is set to 6.3 for this data set. On the other hand, the RPF algorithm is less sensitive to this input parameter, which makes it suitable and more robust for high-throughput unsupervised data analysis.

### 5.5. Pre-calculation of global threshold   

**Figure 15:**
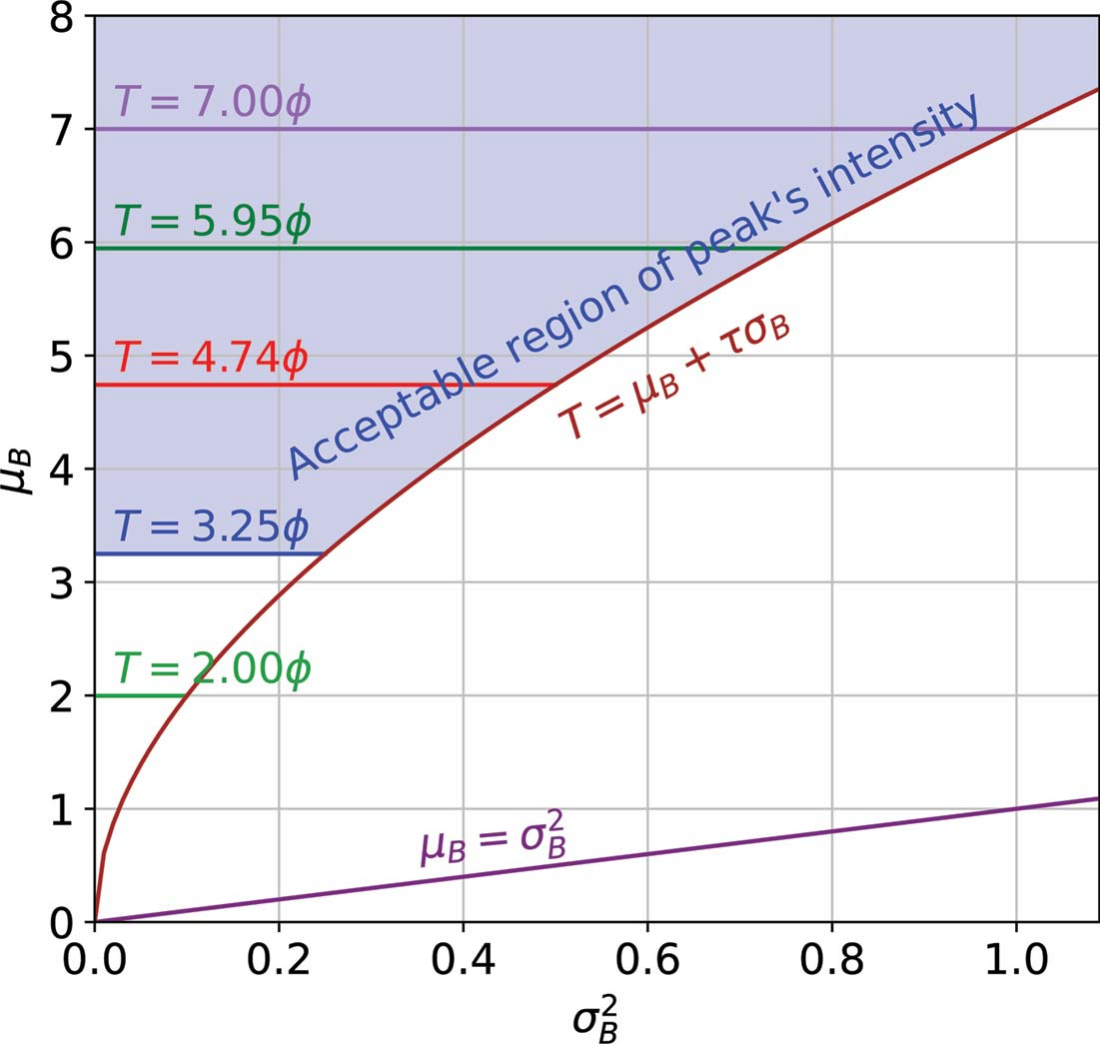
Examples of pre-calculated global thresholds for Bragg peaks using the relation between the average (μ_B_) and variance (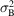) of the Poisson distribution, employed to model the background. The blue region shows the acceptable intensity values for Bragg peak pixels for *T* = 3.25ϕ and τ = 6. The axis values are normalized by ϕ.

Most peak finders have an input parameter for a global threshold for intensity of Bragg peaks. For example, PF8 has an input parameter called ‘threshold’ that allows the user to enter a global value below which Bragg peaks are discarded. Currently, the RPF algorithm does not support such an input. Rather, it pre-calculates this value during a calibration step by using the standard deviation of the detector dark field, σ_D_, and the ADU value (analogue-to-digital units) for a single photon, ϕ. When the estimated background for a Bragg peak is μ_B_ < λσ_D_, the average μ_B_ is dominated by the noise of the detector. Such an estimate is not informative enough and, unless the Bragg peak is very bright, it is rejected. Instead of using the threshold to reject Bragg peaks, RPF uses it to disregard the estimation of the SNR when the background average is too low. The pixel intensity must be above *T* = λσ_D_ + λ(λσ_D_ϕ)^1/2^. In the robust statistics literature, typically 2 < λ < 4. In order to detect the weaker Bragg peaks in diffraction data sets ideally the global threshold should be kept as low as possible. Therefore, the default value for the global threshold was set to λ = 2 (the minimum value recommended in the literature). Knowing that for a Bragg peak the intensity must be above *T* = μ_B_ + τσ_B_, and that in an ideal situation we have 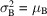, the region of acceptable values for Bragg peaks is shown in Fig. 15 ▸. For the AGIPD-1M high-gain memory cell number 1, we calculated ϕ = 73.5 and σ_D_ = 9.1 (it is expected that σ_D_ = ϕ/6 to separate two Gaussians of zero and one photon by 6σ_D_), which gives *T* = 3.25ϕ. This value is used as the example threshold in Fig. 15 ▸ to show the region of acceptable intensities for Bragg peaks.

For photon-integrating detectors such as AGIPD-1M, σ_D_ can be calculated. For photon-counting detectors (*e.g.* PILATUS 6M) a digital signal is returned, giving the number of photon events counted within the counting time. Photon events are usually detected when the current in the sensor exceeds half the maximum expected for a given photon energy. In such detectors, calculation of σ_D_ is not possible from the output, and we propose σ_D_ = 1/6 as above.

## 6. Conclusion   

In this paper we have introduced an algorithm, termed the ‘robust peak finder’, for outlier detection to identify crystal diffraction patterns in serial crystallography experiments. The algorithm is based on robust statistical methods. We have described a framework with application to serial crystallography data analysis, which is a particularly data intensive field. This algorithm uses robust statistical methods to reduce the number of input parameters and avoid the need for *a priori* knowledge of the experiment. We have shown that the RPF method is effective and extracted a greater number of Bragg peaks from a series of test data sets than previous approaches using the default settings. The results of the data analysis using this method appear reasonable and did not necessitate any fine tuning of the input parameters.

Inevitably, one spends more time optimizing parameters for one’s own algorithm, and optimized parameters for one data set may or may not work for some other data set from a different beamline, different detector, different sample delivery, different crystal quality *etc*. A parameter-free peak finder may never perform quite as well as one optimized by hand for a particular data set, but it may be more useful if it eliminates the need for a time-consuming manual optimization.

We compared the proposed algorithm with the existing state-of-the-art algorithm for different data sets collected under different experimental conditions and found a significant increase in performance in terms of processing time and hit-finding accuracy. This development is important for two reasons. Firstly, it allows for data reduction to be conducted in real time with confidence, meaning the data can be reduced before they are written to file. Secondly, the simplicity of the algorithm makes it more accessible for the general user community, as it requires much less specialist domain knowledge about hit-finding parameters due to the reduction in tunable parameters. We provide a software library containing an implementation of this algorithm which can be easily integrated into any data analysis pipeline and an implementation in the popular crystallographic software libraries *CrystFEL* and *Cheetah*. This work represents a significant step towards fast automatic data processing for serial crystallography experiments performed at high-repetition-rate X-ray sources.

## Figures and Tables

**Table 1:** An overview of the three data sets used for testing the performance of RPF CXIDB32 data set information: Zhou *et al.* (2016 ▸). EuXFEL data set information: Kirkwood *et al.* (2021 ▸). Petra II p11 data set: Oberthuer* et al.* (2016 ▸).

Data set name	Sample	Injection	Beamline	Detector	Space group	*a*, *b*, *c * (Å)	α, β, γ (°)	Photon energy (keV)	Detector distance (cm)
CXIDB32	Rhodopsin–arestin complex	LCP	CXI, LCLS	CSPAD	*P*2_1_*P*2_1_*P*2_1_	109.2, 109.2, 452.6	90, 90, 90	9.5	10
EuXFEL commissioning	Lysozyme	GDVN	SPB/SFX, EuXFEL	AGIPD	*P*4_3_*P*2_1_*P*2	79.20, 79.20, 37.80	90, 90, 90	9.3	11.96
Petra III p11	Dioxygenase	Kapton tape drive	p11, Petra III	PILATUS	*P*2_1_*P*2_1_*P*2	111.51, 154.84, 101.95	90, 90, 90	12	25.2

**Table 2:** Overview of the results for the three different data sets, CXIDB32, EuXFEL commissioning and Petra III p11 Two peak-finding algorithms were tested, RPF and PF8. *CrystFEL* was used to generate the statistics in the table. Values for *R*_split_, {\rm CC}^{*}, *CC*_1/2_ and *I*/σ(*I*) are the overall values reported. Values for the high-resolution shell are given in parentheses.

	No. of hits	Hit fraction (%)	Indexed frames	Indexing fraction (%)	CC_1/2_	*R*_split_ (%)	{\rm CC}^{*}	*I*/σ(*I*)	Resolution range (Å)	Redundancy
Data set: CXIDB32										
RPF	58 695	1.45	54 359	92.61	0.9498	56.06	0.9870	2.81 (0.17)	12.93–3.03 (3.14-3.03)	1470.1 (16.4)
PF8	22 462	0.55	21 875	97.39	0.9127	60.51	0.9769	2.51 (0.09)	12.08–3.01 (3.12-3.01)	1019.4 (7.2)
PF8 on RPF output	–†	–†	36 369	61.96	0.9052	68.71	0.9748	2.68 (0.14)	12.28–3.01 (3.14-3.01)	1208.4 (12.4)

Data set: EuXFEL commissioning										
RPF	2 127 935	37.69	1 742 777	81.90	0.9986	4.52	0.9997	21.04	7.03–1.51 (1.60-1.51)	26545.6 (30.0)
PF8	3 422 532	60.63	1 257 048	36.73	0.9986	4.49	0.9997	19.89 (0.05)	7.08–1.51 (1.63-1.51)	28210.6 (24.9)
PF8 on RPF output	–†	–†	1 663 851	78.19	0.9986	4.50	0.9996	20.81 (0.22)	7.05–1.51 (1.60-1.51)	22743.1 (20.8)

Data set: Petra III p11										
RPF	55 748	12.30	26 346	47.26	0.9740	26.99	0.9934	4.25 (0.36)	23.67–2.85 (2.87-2.85)	64.9 (5.1)
PF8	453 231	100	23,864	5.26	0.9643	30.62	0.9909	3.76 (0.08)	24.40–2.85 (2.86-2.85)	47.3 (2.7)
PF8 on RPF output	–†	–†	21 526	38.61	0.9643	30.62	0.9909	3.74 (0.13)	24.40–2.85 (2.86-2.85)	51.2 (3.6)

† All of the hits found by RPF were fed into PF8 with the hit threshold set to zero.
